# Two different classes of co-occurring motif pairs found by a novel visualization method in human promoter regions

**DOI:** 10.1186/1471-2164-9-112

**Published:** 2008-03-01

**Authors:** Katsuhiko Murakami, Tadashi Imanishi, Takashi Gojobori, Kenta Nakai

**Affiliations:** 1 Integrated Database Group, Japan Biological Information Research Center (JBIRC), Japan Biological Informatics Consortium, Aomi 2-41, Koto-ku, Tokyo, 135-0064, Japan; 2 Integrated Database Group, Biological Information Research Center (BIRC), National Institute of Advanced Industrial Science and Technology (AIST), Aomi 2-41, Koto-ku, Tokyo, 135-0064, Japan; 3 Center for Information Biology and DNA Data Bank of Japan, National Institute of Genetics, 1111 Yata, Mishima, Shizuoka 411-8540, Japan; 4 Department of Genetics, The Graduate University for Advanced Studies, 1111 Yata, Mishima, Shizuoka 411-8540, Japan; 5 Human Genome Center, The Institute of Medical Science, The University of Tokyo, Shirokanedai 4-6-1, Minato-ku, Tokyo, 108-8639, Japan; 6 Institute for Bioinformatics Research and Development (BIRD), Japan Science and Technology Agency (JST), Japan

## Abstract

**Background:**

It is essential in modern biology to understand how transcriptional regulatory regions are composed of *cis*-elements, yet we have limited knowledge of, for example, the combinational uses of these elements and their positional distribution.

**Results:**

We predicted the positions of 228 known binding motifs for transcription factors in phylogenetically conserved regions within -2000 and +1000 bp of transcriptional start sites (TSSs) of human genes and visualized their correlated non-overlapping occurrences. In the 8,454 significantly correlated motif pairs, two major classes were observed: 248 pairs in Class 1 were mainly found around TSSs, whereas 4,020 Class 2 pairs appear at rather arbitrary distances from TSSs. These classes are distinct in a number of aspects. First, the positional distribution of the Class 1 constituent motifs shows a single peak near the TSSs, whereas Class 2 motifs show a relatively broad distribution. Second, genes that harbor the Class 1 pairs are more likely to be CpG-rich and to be expressed ubiquitously than those that harbor Class 2 pairs. Third, the 'hub' motifs, which are used in many different motif pairs, are different between the two classes. In addition, many of the transcription factors that correspond to the Class 2 hub motifs contain domains rich in specific amino acids; these domains may form disordered regions important for protein-protein interaction.

**Conclusion:**

There exist at least two classes of motif pairs with respect to TSSs in human promoters, possibly reflecting compositional differences between promoters and enhancers. We anticipate that our visualization method may be useful for the further characterisation of promoters.

## Background

The transcription of genes is considered to be primarily regulated by transcription factors (TFs). TFs are recruited to interact with specific DNA sequences or motifs in promoters. It is an important and challenging issue to characterize these promoter motifs. It was shown that some motifs (e.g., TATA, SP1, and CREB) are most commonly found within -400 to +100 bp of the transcription start site (TSS) [1]. In other words, positional distribution plots of single motifs found in many promoters show a single peak in frequency near the TSS [1,2]. In the promoters of higher eukaryotes, such as humans, multiple TFs act in a coordinated way to enable complex patterns of gene expression; thus, it is highly likely that even those binding sites of TFs that do not show clear positional preferences are placed under some constraints, showing a bias such as a frequency peak at a certain distance from the TSS when the co-occurrences of a motif pair are considered. To date, no study has examined the positional relationships among motif pairs and TSS. Furthermore, the search for motifs is usually limited to the 1 kb of sequence immediately upstream of a TSS; however, more than 1 kb of upstream sequence is commonly conserved among vertebrates. A previous study has reported a diverse range of different TSSs for the same genes [3], and distal-promoters are also important for gene expression [4]. Therefore, > 1 kb of sequence should be examined for motif pairs.

In higher eukaryotes, TF binding sites are often organized in clusters called cis-regulatory modules (CRM) [5]. Most of the computational CRM prediction algorithms developed to date rely mainly on the distances between motifs and the phylogenetic conservation of these motifs across different species [6-8]. For example, Blanchette et al. recently performed a genome-wide prediction of CRMs in the human genome [8]; however, further analyses are required to clarify the nature of different motifs, such as which motif pairs are preferentially used in promoters. Yu et al. [9] addressed this issue by identifying motif pairs with biased distance distributions in various sets of human genes that exhibited tissue-specific expression, and Long et al. [10] identified motif pairs over-represented in promoters of immune-response genes. It also appears to be important to gain an understanding of the function of upstream sequences that are not specific to differentially expressed genes. Such overall analyses may reveal the specific features of (a partial set of) house-keeping genes.

We are currently engaged in the construction of two databases (DBTSS [11] and H-InvDB [12]) that contain information on TSSs determined by the mapping of cDNAs with intact 5'-ends onto genome sequences. H-InvDB covers a wide range of genes, but the TSS information is expected to be more accurate in DBTSS. This wealth of data should be useful in obtaining further insights into motif positioning relative to TSSs.

In this study, we developed a method of identifying positional relationships among motif pairs and TSSs in human promoters. We searched from -2000 to +1000 bp relative to each TSS, enabling an examination of possible alternative promoters [13] or distant motifs in enhancers and silencers. We first visualized the positional preferences among pairs of non-overlapping motifs and the representative TSS for each gene. This visualization assisted in the identification of two distinct classes of motif pairs in terms of the positional relationships between the pairs and TSSs. Differences between these two classes were examined with regard to the positional distributions of their constituent motifs, the expression patterns of the genes that harbor the motif pairs, and the protein domains relevant to the transcription factors that bind to the promoter motifs.

## Results

### Identification and visualization of co-occurring motif pairs with positional correlation

We predicted the DNA motifs, or putative transcription factor binding sites, in the sequence from -2000 to +1000 bp relative to the TSSs of human genes using 228 weight matrices in the TRANSFAC database. For convenience, we refer to these as 'promoter sequences'. The weight matrices were clustered such that each cluster of weight matrices can be regarded as non-redundant (see the Methods section). To reduce the number of false positives (non-functional sites), we discarded motifs predicted on repetitive sequences such as Alu and those predicted outside of phylogenetically conserved regions. The remaining putative sites were used for analyses of their co-occurrence. To avoid counts based on overlapping binding motifs, overlapping pairs were not considered. The chi-square test was used to identify motif pairs that co-occur with significant positional correlation; i.e., pairs for which the bias of the co-occurrence is not explained by the independent occurrence of each motif. The motifs were analyzed using Kolmogorov-Smirnov test (K-S test) to assess the significance of their distance distributions relative to those of randomly generated sequences. A criterion of 1% false discovery rate (FDR) was applied to both the chi-square test and the K-S test. Motif pairs that satisfied the two tests were considered to be significant.

**Figure 1:**
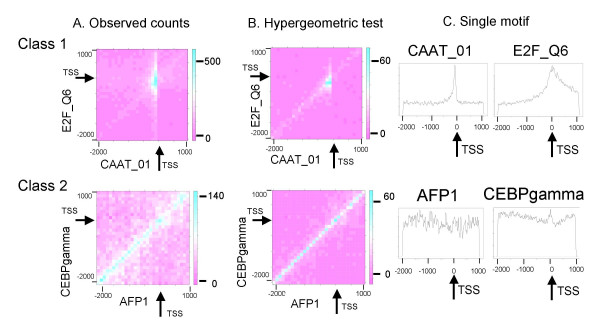
Visualization methods for illustrating the positional preferences of motif pairs with respect to TSSs. Regions [-2000, +1000] relative to TSSs are regarded as 'promoters' (TSS positions are indicated by arrows in the Class 1 examples; for an explanation of the different classes, see the main text). A motif pair, that of CAAT_01 and E2F_Q6, is shown as an example of the Class 1 motif pairs, while a second pair, that of AFP1 and CEBPgamma, is used as an example of Class 2 pairs. (A) Heat map showing the frequency of the motif pair. Each region, which represents a region-pair, is colored according to the raw counts of the motif pair considered. High counts are shown in red, intermediate counts in white, and low counts in blue. The presence of CAAT_01 (upper figure) and AFP1 (lower figure) are shown on the X-axes for the two examples. (B) Heat map showing the negative logarithm of *p*-values calculated for the significance of positional preference. (C) Positional distribution of a single motif on promoters. The Y-axis indicates the frequency of the motif at each position represented in the X-axis in all promoters.

Among the 26,106 motif pair combinations, 8,454 (32%) and 5,668 (22%) were identified as being significant in terms of the promoters from H-InvDB and DBTSS, respectively. All of the significant motif pairs are listed in Additional file 1. To examine the patterns of their positional biases, we visualized the co-occurrences of motif pairs in three ways. First, we compiled a heat map of the raw counts of each motif pair along the promoter regions (Figure 1A). Second, we plotted another heat map showing the negative logarithm of *p*-values for the significance of the bias, calculated assuming a hypergeometric distribution for each region-pair (Figure 1B). In other words, this plot shows the degree to which the co-occurrence is enhanced at a region-pair compared with an independent occurrence at the same bin. Third, we plotted the positional distribution of each motif on each promoter (Figure 1C). Using this approach, we visualized the biased occurrences of both paired and single motifs. This analysis revealed a number of common patterns that have interesting biological implications.

### Comparison of the detected motif pairs and known data

For the significant motif pairs, we examined the 'detection ratio', which is the ratio of the number of detected and known motif pairs to the number of known motif pairs, using the TRANSCompel database (a database of composite promoter elements [14], ver. 9.2) and the TRANSFAC database. Since the definition of identical motifs (grouping of motif matrices) in TRANSCompel database is different from ours (i.e., we used more stringent criteria for motif clustering), the correspondence of neither the motifs nor the motif pairs is 1-to-1. Of the 8,454 significant motif pairs, 547 were successfully mapped to 86 of the 127 pairs in TRANSCompel. Thus, the detection ratio of TRANSCompel was 69% (86/127).

### Classification of motif pairs based on positional patterns

**Figure 2:**
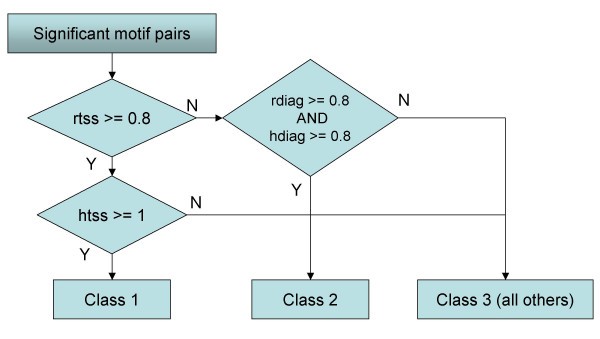
Flow chart employed in defining the three classes of motif pairs. See "Classification of motif pairs" in the Methods section, for definitions of 'rtss', 'rdiag', 'htss', and 'hdiag'. 'Y' and 'N' indicate 'yes' and 'no', respectively.

Visualization of the two types of heat maps shown in Figure 1A and 1B enabled the identification of several characteristic groups. To objectively analyze the data from these heat maps, we defined a set of criteria to use in classifying motif pairs into three groups; a flow chart of this process is shown in Figure 2. Briefly, in the Class 1 pairs, co-occurrences are found mainly in the vicinity of TSSs [-500 to +500] in both types of heat maps. In the Class 2 pairs, co-occurrence was not particularly biased around TSSs, but was biased when the relative distances of co-occurring positions were within 500 bp in both heat maps. Those motif pairs not classified as Class 1 or 2 were assigned to Class 3, which contains miscellaneous bias patterns. We emphasize that this classification of motif pairs is based solely on plots of raw counts (Figure 1A) and the hypergeometric test (Figure 1B): it does not take into account the positional distribution of single motifs (Figure 1C).

The numbers of motif pairs classified into Classes 1, 2, and 3 were 248, 4020, and 4186, respectively. Figure 1 shows typical examples of Class 1 and Class 2 pairs. As noted above, Class 2 pairs occur over a wide range (within the 3 kb range included in our analysis) relative to the positions of TSSs. In most cases, these motif pairs were located within 100 bp of the TSSs, which is the unit length of our digitization (*L*_*b*_) (see Figure 1, and the Methods section).

### Types of motif peaks in terms of the positional distributions of single motifs

In some cases, the positional distribution of a single motif exhibits a peak around the TSS ([1,2]; Figure 1C); thus, we characterized the above co-occurrence patterns (Figure 1A and 1B) in terms of the positional distributions of their constituent single motifs. Based on the signal/noise ratio of the positional distribution, we detected peaks within -300 to +300 of TSSs (for a detailed description, see the Methods section). Similar peak-detection methods, employing standard deviation, have been used in previous studies [1,15]. Each single motif was assigned into one of four peak types: 'Large Peak (LP)', 'Small Peak (SP)', 'No Peak (NP)', and 'No Data (ND)'. Among the 228 independent motifs (or motif clusters), we classified 56, 49, 100, and 23 into types LP, SP, NP, and ND, respectively.

**Figure 3:**
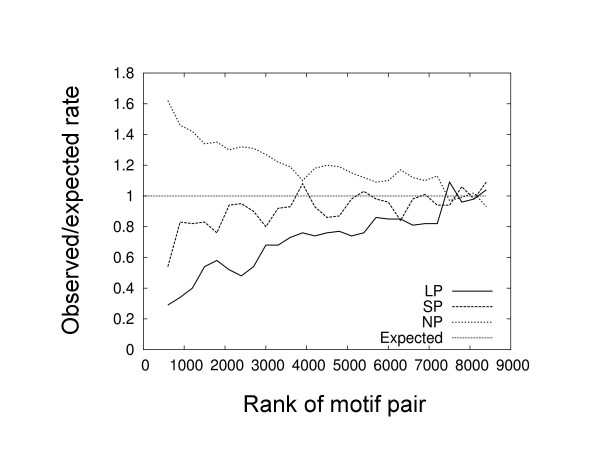
Preferences of the peak types versus the ranks of the significance of co-occurrence. The Y-axis represents the ratio of observed to expected numbers of motifs found in groups of 300 motif pairs ranked according to co-occurrence. At X = 600, values for the top 600 pairs are shown.

We examined the relationship between the co-occurrence of motif pairs and the type of peak of their constituent motifs based on the rank of *p*-values calculated for the significance of motif pairs, as assessed using the chi-square test (Figure 3). For every group of 200 pairs (in descending order of this ranking), the content of each peak was counted and its ratio compared with its expected count. We were unable to rank the top 544 pairs because their *p*-values were close to zero; consequently, the values of the top 600 pairs were plotted as the first group (the group size was 200 pairs thereafter). It is evident from Figure 3 that the NP motif type is strongly preferred in higher ranks of motif pairs: the LP and SP types tend to be avoided.

Next, we considered the relationship between the different peak types and the classes of motif pairs. Table 1 lists the 20 motifs that are most frequently used in the motif pairs in each class. These motifs (or transcription factors that bind to these motifs) can be regarded as the 'hubs' in the network. For convenience, we refer to the top 20 motifs as hub-motifs (hub-TFs). A log-log plot (Additional file 2) reveals that the degree (the number of motif counterparts) in Class 2 decreases linearly as the rank number increases, while that in Class 1 decreases rapidly. This finding suggests that the connectivity of the two classes is intrinsically different.

**Table 1:** Top 20 motifs included in the two motif-pair classes, and all significant pairs.

	Class 1			Class 2			Total		
	
Rank	Motif	Count	P	Motif	Count	P		Count	P
1	SP1_Q6	37	LP	NMYC_01	129	SP	GATA4_Q3	202	NP
2	E2F_01	29	LP	GATA4_Q3	128	NP	CEBPGAMMA_Q6	197	NP
3	MAZ_Q6	29	LP	TFIIA_Q6	115	NP	NMYC_01	191	SP
4	NFY_Q6	23	LP	FOXP3_Q4	114	NP	CDPCR1_01	186	NP
5	KROX_Q6	20	LP	CEBPB_01	110	NP	EVI1_04	183	NP
6	PAX3_B	20	LP	PBX_Q3	108	NP	NFKAPPAB_01	182	SP
7	SP3_Q3	18	LP	CDX2_Q5	108	NP	CDX2_Q5	181	NP
8	CP2_01	17	SP	CEBPGAMMA_Q6	106	NP	TFIIA_Q6	181	NP
9	ELK1_01	16	LP	EVI1_04	106	NP	E2F_01	179	LP
10	ATF_01	14	SP	CDPCR1_01	106	NP	MAZ_Q6	177	LP
11	MAZR_01	13	LP	FOX_Q2	105	NP	FOX_Q2	177	NP
12	MINI20_B	13	LP	NKX25_01	103	NP	CEBPB_01	176	NP
13	NMYC_01	12	SP	OCT1_Q6	102	NP	PBX_Q3	175	NP
14	NFKAPPAB_01	12	SP	MEF2_Q6_01	101	NP	MEF2_Q6_01	172	NP
15	PAX9_B	12	LP	GATA1_02	101	NP	TBX5_02	171	NP
16	HES1_Q2	10	SP	HNF1_Q6	99	NP	HNF1_Q6	171	NP
17	ATF1_Q6	10	LP	CEBPDELTA_Q6	98	NP	EFC_Q6	170	SP
18	RFX1_02	9	LP	CDC5_01	97	NP	FOXP3_Q4	167	NP
19	YY1_02	8	LP	POU1F1_Q6	93	NP	MYOD_01	165	SP
20	AP2ALPHA_02	8	LP	NKX25_02	92	NP	SP1_Q6	164	LP
21	SMAD4_Q6	8	SP	HNF3B_01	92	NP			
22	VMYB 01	8	SP						

The actual number of motifs exceeds 20 because of tied ranks. The identifiers in TRANSFAC are shown as the names of the motifs. The 'PT' column shows the peak type of each motif (LP: large peak, SP: small peak, and NP: no peak).

Interestingly, most of the peaks in Class 1 were LP (and SP), whereas those in Class 2 were mainly NP. This indicates that many of the constituent motifs in the Class 1 pairs occur around TSSs; however, the predominance of LP-type peaks in Class 1 is not the result of an independent preference for localization near TSSs: instead, it reflects cooperation between pairs. This interpretation is supported by the significant peaks in the *p*-value heat map (Figure 1B). Another interesting observation is that the hub motifs in the two classes define almost completely disjoined sets (the only exception being motif 'NMYC_01'; see Table 1). In contrast, if we compare all of the motifs (i.e., both hubs and non-hubs) that appear in the two classes, they show significant overlap. There are very few motifs specific to Class 1 (data not shown).

### Promoters that contain Class 1 motif pairs prefer ubiquitous expression

**Figure 4:**
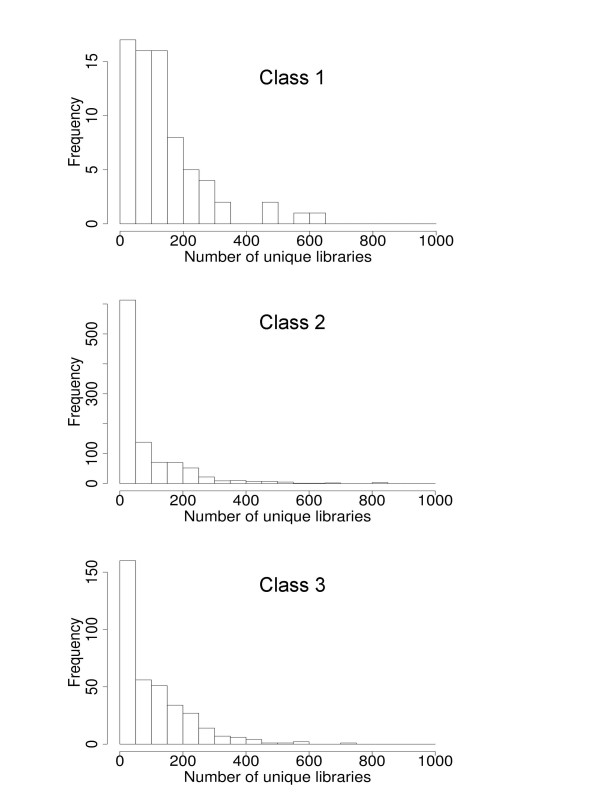
Distributions of the number of unique EST libraries for the genes related to each class. The X-axis represents the number of different EST libraries in which the expression of a gene is observed, which is regarded to be a measure of ubiquitous expression. The Y-axis represents the frequency of genes corresponding to the bin of the X-value.

It is of interest to test for differences between the expression of genes that posses the two classes of motif pairs. To this end, we used the UniGene database [16] in assessing the tissue-specific/ubiquitous expression of genes. As exemplified in the work by Yamashita et al. [17], the number of unique EST (Expression Sequence Tag) libraries in which expression of a gene is observed as at least one EST sequence can be used as a convenient measure of anti-tissue specificity. To examine gene expression, we prepared a gene set putatively regulated by the motif pairs in each class. As each promoter often contains multiple motif pairs from multiple classes, it is not simple to connect each class to either gene or gene expression. Here we collected genes whose promoters contain only motif pairs in the same class (see the Methods section). Each group contained 72, 1011, and 364 promoters for each class. Figure 4 shows the distribution of the number of different EST libraries for each class. The mean numbers of different libraries for Classes 1, 2, and 3 are 137.7, 75.1, and 104.3, respectively. The *p*-value on the null hypothesis that there is no difference between Class 1 and Class 2 (Class 3) was lower than 9.4e-10 (6.4e-3) by the Wilcoxon test. We also calculated correlation coefficients between the number of motif pairs in each class and expression measure (logarithm of the unique number of libraries for each gene) using all genes. They were 0.23, -0.12, 0.03 for each class. It seems evident that those genes that possess Class 1 motif pairs on their promoter regions are more likely to be expressed ubiquitously than those that possess other classes of motif pairs.

### Relationships between the class of motif pairs and CpG richness of promoters

We considered the relationship between the classes of motif pairs and the CpG richness of related promoters. We calculated CpG ratio in the non-repeat sequences of promoters as an index of CpG richness. The number of CpG in all the regions, including repeat sequences (*e.g*. Alu), gave similar results. The correlation coefficient between the number of motif pairs from each class and the CpG ratio through all promoters were 0.38, -0.40, and 0.02, respectively. Both positive and negative significant correlations were observed for Class 1 and 2. There were no significant correlation between the CpG ratio and Class 3. Assuming that motif pairs in Class 1 are more related to ubiquitous gene expression, the results of Class 1 and 2 are consistent with the fact that about half of housekeeping genes have CpG islands covering the TSS [18].

### Protein domains characteristic of the hub-TFs

Using the 600 most significant motif pairs, we constructed a network of motif pairing. A log-log plot (Additional file 3) of the degrees-node distribution showed linearity, suggesting that the network obeys the power law, as is typical for protein-protein interaction networks [19]. As noted above, those TFs that correspond to the top 20 motifs in each class in Table 1 are regarded as hubs in the network. To characterize these TFs, their domain structure was examined using the TRANSFAC database. Of the 228 TFs linked to the 600 motif pairs, 188 possess 664 domains. Among these domains, enriched domains with odds ratios above 1.5 are listed in Table 2 (see the Methods section). The odds ratio indicates the degree to which the domain is enriched in the hub-TFs. The data in Table 2 reveal that domains rich in certain amino acids (e.g., glutamine/proline) are enriched in Class 2 hub-TFs and the entire set of significantly co-occurring pairs.

**Table 2:** Domain analysis of transcription factors.

Domain name	Occurrence of domain	# of TF	Odds ratio	P-value
	Class 1			
	
serine-rich	55	3	3.00	3.52E-13
dimerization	86	6	2.46	1.86E-15
basic	82	5	2.24	1.73E-12
helix	81	5	2.12	5.87E-11
HLH	51	3	2.04	1.11E-06
zinc_finger	151	8	1.89	6.16E-18
leucine_zipper	65	4	1.70	1.97E-05
glutamine-rich	85	6	1.65	1.90E-06
trans_activation	91	6	1.61	2.12E-06

	Class 2			
	
glutamine-/proline-rich	335	3	2.85	4.65E-65
serine-rich	344	3	2.66	5.85E-60
alanine-rich	321	3	2.48	2.22E-49
proline-rich	551	5	1.73	8.47E-39
POU-domain	294	3	1.67	1.98E-17
serine-/threonine-rich	524	5	1.65	3.26E-31
leucine_zipper	443	4	1.64	3.56E-25
forkhead	311	3	1.55	1.65E-14

	All			
	
serine-rich	723	4	3.46	3.29E-187
glutamine-/proline-rich	540	3	2.85	1.20E-103
proline-rich	1,419	8	2.77	7.95E-302
glycine-rich	885	5	2.59	2.51E-153
alanine-rich	533	3	2.55	2.14E-85
dimerization	893	5	2.24	3.48E-118
helix	876	5	2.01	1.98E-90
HLH	535	3	1.88	3.08E-44
basic	711	4	1.70	4.24E-45
leucine_zipper	743	4	1.70	2.65E-47
serine-/threonine-rich	869	5	1.70	4.98E-56

Shown are the domain names, the frequency of the domains in the hub-TFs (i.e., the TFs in Table 1) for class 1, 2, and all of co-occurring motif pairs. The domains are ordered by odds ratio.

## Discussion

We identified TRANSFAC motif pairs that significantly co-occur within human promoter regions. This procedure is dependent on the accuracy of the TSS information. For TSS annotation, we used two databases: H-InvDB and DBTSS. H-InvDB contains more sequence data of transcripts that cover not only protein-coding mRNAs but also Pol II-transcribed non-coding RNAs. Because the promoter sequences are more abundant in H-InvDB, fewer false negatives are expected; it is therefore natural that a greater number of significant motif pairs were found in H-InvDB than in DBTSS. On the other hand, the advantage of using DBTSS lies in the accuracy of its TSS information. Given that we confirmed that the co-occurrence patterns for motif pairs derived from both annotations were similar, we used H-InvDB for down-stream analyses.

The detection ratio of significantly detected motif pairs was 69% (86/127), while we detected 32% of the total possible combination (i.e. 8,454 motif pairs out of 228 × 227/2 motif pairs), which is a promising result considering the previous work by Yu et al. [9]. Its detection ratio (or sensitivity) was 40%, detecting 19% (9,060 motif pairs) of 46,971 combination using tissue specific gene groups. Our detection ratio is higher than that of Yu et al., mainly because we detected many pairs. Related to the reason of our high detection ratio, the substantial difference is that we use all genes transcribed by RNA pol II including widely expressed (housekeeping) genes, while they only use tissue specific genes. Other differences include the evaluation model, source of known interactions (they used DIP [20] and TRANSFAC), and clustering of motifs. For the evaluation model, we evaluated motif pairs with two statistical tests: (1) chi-square test for biases of their relative positions from TSS and (2) K-S test for their distance distribution biases. Yu et al. evaluated motif pairs from two viewpoints: (1) over-representation of motif pairs and (2) distance distribution biases, which we adopted as the second test. Thus, their model does not consider the distance between the motif and TSS. They evaluated a p-value as the product of the two p-values calculated from the above viewpoints. For the thresholds, we used an FDR of 1% for each test, though 1%, 5%, and 10% are conventionally used [21,22]. Yu et al. set the threshold to be 10^-6.2 ^after the multiple testing correction. If we calculate the product of the p-values corresponding to the two tests, their maximum value among the detected motif pairs was 5.9 × 10^-5^. In addition, if we employ the same threshold of 10^-6.2^, 8,379 out of 8,454 pairs were still significant. Thus, our threshold of FDR is stringent enough.

A total of 8,454 pairs were analyzed for their positional preference with respect to the TSS position; consequently, two major classes were identified. The visualization of the bias of motif pairs in terms of the promoter (Figure 1A and 1B) is a novel approach that enables the identification of Class 2 pairs. In the hypergeometric statistical test shown in Figure 1B, if the tested region lies only on the core promoter, it is the same as those used in several previous papers [7,23]. We extended the single region to wider set of regions for the evaluation of co-occurrences for motif pairs on divided promoter region pairs (bin pairs) to detect positional correlations. The approach taken in this regard – to plot a positional distribution for single motifs and to identify a peak (see Figure 1C) – is widely used in previous studies [1,2]. We then defined a set of criteria to employ in objectively assigning pairs to different classes (Figure 2). Several features of these two classes of motif pairs were then examined, with the results being summarized in Table 3.

**Table 3:** Summary of the features of co-occurring motif pairs.

Feature	Class 1	Class 2	Class 3
Number of pairs	248	4,020	4,186
Pattern on the heat maps	Around TSS	Scattered diagonally	uncharacterized
Dominant peak types of single motifs	LP/SP	NP	NP
Correlation with CpG ratio	0.38	-0.40	0.02
Expression preference	Ubiquitous	Specific	Specific
Typical domains of corresponding TFs	Dimerization, Basic, Alpha-helical, [SG]-rich	[APSTQ]-rich	miscellaneous

LP, SP, NP are the different peak types for single motifs (LP: large peak, SP: small peak, and NP: no peak).

The Class 1 motif pairs are localized near the TSSs, and may represent components of core promoters [1,24]. These motif pairs mainly consist of LP and SP types of single motifs, which is notable for two reasons. First, in significantly co-occurring motif pairs, NP types of single motifs are overrepresented (Figure 3). Second, in the heat map that shows the significance of correlated co-occurrence (Figure 1B), the overrepresentations of the co-occurrence of the motif pairs cannot be explained as a direct result of the independent occurrence of each motif around the TSS. Thus, it is possible that the TFs that bind to these motif pairs interact with each other.

The possibility that the Class 1 motif pairs are largely related to constitutive or ubiquitous gene expression is indicated by the three following evidences: (1) the EST analysis (Figure 4), (2) the functions of the TFs that binds to the top 20 motifs that are most frequently used in the Class 1 pairs, and (3) a recent work conducted by another group [25]. For example for the functions of the TFs, the transcription factor E2F regulates gene expression in the cell cycle; SP1, MAZ, NFY, KROX, SP3, and CP2 are the TFs associated with ubiquitous transcription; and PAX3 (rank number 6) is a member of the paired box (PAX) family of transcription factors, that has multiple roles during fetal development. As the third evidences, according to a recent study on alternative promoters [25], single promoters that are CpG-rich and do not have alternative promoters are significantly associated with ubiquitous gene expression. It is likely that this category of promoters is mainly composed of our Class 1 motif pairs.

The relative positions of motif pairs are much more flexible in Class 2 than in Class 1. These pairs appear at least 3 kb away from the TSS, yet most previous studies only examined 1 kb of sequence [26]. These Class 2 motif pairs are mainly composed of NP-type motifs, which are likely to cooperate with other motifs to be functional. In *S. cerevisiae*, Yu et al. [27] found 300 significant TF interactions (as motif pairs), most of which were not constitutively active. This is consistent with our study of human promoters because the motifs related to constitutive gene expression are thought to be linked to Class 1, which is the minority of the detected motif pairs. Moreover, the top 20 most frequent motifs in Class 2 (as shown in Table 1) appear to be involved in various functions. We show some examples of TF pairs, corresponding to detected motif pairs, that are known to function cooperatively. We detected a motif pair consisting of GATA4 and AP1. GATA4 is thought to regulate genes involved in embryogenesis and in myocardial differentiation and function, and GATA4 and AP1 cooperatively regulate transgenic mice overexpressing cardiac calsequestrin [28]. We have another motif pair consisting of GATA4 and TBX5. TBX5 plays a role in heart development and specification of limb identity together with GATA4 [29]. One of our hub motifs MYOD pairs with several motifs known to be involved in muscle and heart gene regulation (MEF2, SRF, SRF, and AP1) [30]. The motif pair NF-kappaB and C/EBP was also detected, and it is known that these two motifs synergistically regulate the mouse serum amyloid A gene expression induced by inflammatory cytokines [31]. Thus, the motif pairs in Class 2 appear to be involved in various specific functions.

We observed that relative positions of motif pairs in Class 2 ranged widely within the 3 kbp regions. It may be due to alternative promoters [13] or the diversity of TSSs [3]. Kimura et al. [13] showed that at least 52% of human genes have alternative promoter sequences separated by >500 bp. Even with a much more strict threshold of 2 kb, 44% (6,485 of 14,628 genes) were regarded to have alternative promoters (see supplementary Figure 1 of [13]). The other possible reason is that these motif pairs can work at any distance as components of enhancers (*cis*-regulatory modules). It is well accepted that the location of enhancers is rather arbitrary and that both promoters and enhancers share modular architecture [32]. Thus, it seems likely that some promoters contain enhancer-like modules (in addition to the core region) and that these modules contain the Class 2 motif pairs. In any case, the fact that the top 20 motifs in both classes do not overlap (except for a single motif) supports the view that the two classes are distinct in terms of the way they function.

We selected a set of TFs that work as hubs in the TF-TF interaction network in each class. Table 2 summarizes the overrepresented domains in these hubs. It seems that these domains are commonly characterized by an excess of a certain amino acid(s). This tendency is stronger in Class 2-related TFs than Class 1-related TFs. These biased domains, such as the proline-rich domain, may play common important roles in gene regulation. In fact, the SH3 and WW domains recognize proline-rich peptides. The binding of SH3 domains to proline-rich regions causes the formation of a large number of protein complexes [33]. Most of the proteins that interact with SH3 domains contain at least one PxxP motif [34]. It was recently found that 49% of the entire sequence of human TFs contains intrinsically disordered (ID) regions [35] that differ from DNA binding domains. Using techniques such as NMR (Nuclear Magnetic Resonance), it has been revealed that the trans-activation domains are unstructured in unbound TFs and become structured upon binding to their partners [35]. The ID regions tend to consist of alanine, glutamic acid, glycine, lysine, proline, glutamine, arginine, and serine residues [36]. Among these eight amino acids, five are overrepresented in Table 2. Furthermore, Haynes et al. reported that ID regions are common to hub proteins from four eukaryotic interactomes (*C. elegans, S. cerevisiae, D. melanogaster*, and *Homo sapiens*) [37], indicating the importance of ID regions in protein interactions. As an unstructured protein domain with repetitive sequence, the C-terminal domain (CTD) of RNA polymerase II is comprised of a variable number of tandem heptapeptide repeats. This feature is important in the efficient capping, splicing, and polyadenylation of mRNA transcripts [38]. These previous studies lead us to a model in which repetitive domains have potential in terms of acquiring multiple functions through protein interaction. Thus, it is likely that the domains commonly observed in hub-TFs (see Table 2) are related to ID regions; this may play an important role in TF-TF interaction in transcription.

With the intention of identifying general features of promoter architecture, the present study examined a general set of genes rather than a set of co-regulated genes, as done in previous studies [9,10,26]. Further studies that employ this approach will help to clarify these general features. A possible approach would be the identification of *cis*-regulatory modules (CRMs) based on the detected motif pairs in this paper, as attempted in [39]. Given that the unit length employed in the present study was 100 bp, a necessary future study would involve a more detailed analysis of the distances between significant motif pairs. We found that most of the motif pair distances are less than 100 bp, with few in the range of 200 bp. This finding is consistent with the observation that most of the cooperative motifs in the TRANSCompel database (a database of composite promoter elements [14], ver. 9.2) are within a distance of 140 bp. Another necessary future task is a more detailed characterization of the remaining significant motif pairs that we tentatively termed Class 3. It seems certain that our visualization method, as well as our statistical tools, will be useful in obtaining further insights into promoter structures.

## Conclusion

Our results indicate the occurrence of at least two distinct classes of motif pairs in human promoters, with one class appearing around TSSs and the other found at somewhat arbitrary distances from TSSs. This trend is reminiscent of the well-known difference between promoters and enhancers (CRMs). There has been some discussion that promoters and enhancers are alike in terms of their modular structure as clusters of TFBSs [32]; however, a recent study reported differences in terms of the pattern of histone modifications [40]. Thus, our findings may reflect this kind of difference or merely differences between core promoters and CRM-like modules of promoters; further investigations are necessary to clarify these hypotheses. Our visualization method will also be useful in these types of future studies.

## Methods

### Prediction of motifs on human promoters

The sequence between -2000 bp and +1000 bp relative to the TSS of each gene was extracted as its 'promoter sequence' from the human genome sequence (NCBI build 35), based on the annotations from H-InvDB [12] release 3.4 and DBTSS [11] version 5.0. We used 'cluster_start/end' from H-InvDB and 'representative TSS' from DBTSS as the annotation for TSSs. Repetitive sequences were masked by RepeatMasker [41]. A total of 34,562 and 11,682 promoters were obtained from H-InvDB and DBTSS, respectively. The motifs (transcription factor binding sites) were then predicted using the 'match' program [42] provided in TRANSFAC [43] (ver. 9.2). As options, we chose the vertebrates' matrices with 'high quality' and used the cut-off values that 'minimize the sum of both error rates', where 'both errors' means errors related to sensitivity and specificity, or Type I and II errors in statistics. Predicted sites were used on both orientations (plus and minus strands). In the case of self-overlapping sites, the one with the higher score was selected. Only those motifs found in highly conserved regions among multiple species were used. These highly conserved regions were determined by the 'phastCons' score, which is based on a phylogenetic hidden Markov model of 17 vertebrates [44]. The score was taken from the conserved track (file 'multiz17way') in the UCSC genome browser database [45]. The threshold of the score was set to 0.54, which maximizes the difference in the ratio of promoters that are conserved/non-conserved at between -500 and +1 (TSS).

### Evaluation of the positional preferences of motif pairs

**Figure 5:**
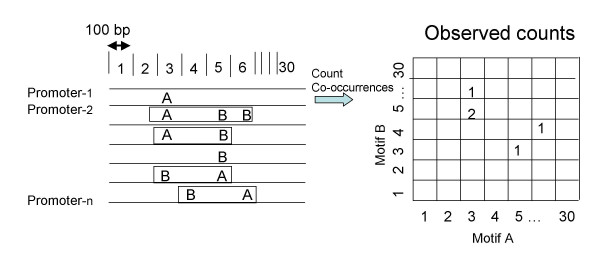
Schematic showing the method employed in counting the co-occurrence of the predicted motifs. Each DNA sequence was divided into *N*_*b *_bins of 30,000/*N*_*b *_(=*L*_*b*_) bp (left; *N*_*b *_and *L*_*b *_are set to 30 and 100, respectively, in this figure). Co-occurrences for each bin-set are identified for each motif set (motifs A and B; sets enclosed in rectangles in the left-hand figure). For example, the count of the co-occurrence of the motif set (A, B) on promoter-2 was two; therefore, in the contingency table, the counts of the cells (A3, B5) and (A3, B6) were increased by one (right). Overlapping motif pairs were not counted. Based on the counts of the co-occurrences in all promoters, we constructed a contingency table for each motif pair.

To assess the positional preferences of co-occurring motif pairs, we split the [-2000, +1000] region into bins of *L*_*b *_bp in length and counted the number of co-occurrences in each pair of bins, making a contingency count table for each pair of motifs (see Figure 5; co-occurring motifs are placed in rectangles in the left-hand figure). The value of *L*_*b *_was determined such that it should be the minimum in a set (100, 150, 200, 250, 300, and 500) where the contingency table satisfies the Cochran rule (no expected cell counts are less than 1 and no more than 20% are less than 5). To avoid bias arising from overlapping motif sequences, only non-overlapping co-occurrences were counted. To compensate for this reduction in the number of overlapping motifs, counts corresponding to uncounted positions were estimated and adjusted. For each pair, the significance of the positional bias at the time of its co-occurrence was tested by the chi-square test on the contingency table and a multiple hypothesis test with a threshold of 1% of false discovery rate (FDR). To further assess the significance of the distance distribution within each pair, we performed a second statistical test against the above significant pairs according to Yu et al. [9]. We generated 34,562 random sequences, each of which has the same base composition as that of each promoter from the H-InvDB annotation; the presence of TRANSFAC motifs on the random sequences was predicted in the same way. For each motif pair, we applied the Kolmogorov-Smirnov Test in comparing the distributions between the two sets of sequences (actual promoters and random sequences). Taking into account the 1% FDR threshold, we selected those motif pairs showing the significance of the biases of the distance distribution. To summarize, motif pairs that showed positional biases with the TSSs (from the chi-square test) as well as biases of their distance distribution (from the K-S test) were chosen as significant.

### Visualization of the positional preference of motif pairs for the purpose of classification

We visualized the co-occurrence using two types of heat maps. The first was a heat map of the raw counts in the contingency table (with adjustments for counts with overlapping cases; see above), as exemplified in Figure 1A. The second was a heat map of the negative logarithm of the *p*-value of the co-occurrence, assuming a hypergeometric distribution for each region-pair (Figure 1B). In the case that the region-pair was located in the same region, the method employed in calculating *p*-values was the same as that employed in earlier studies [7,23]. That is, suppose a region-pair, [-200, -100] (relative to TSS) for motif A and [-1000, -900] for motif B. It is possible to consider a 2 × 2 contingency table based on whether each motif occurs or not. Based on this table, *p*-values are calculated for each pair using the following formula based on the assumption of no positional preference:

$$P_{hg}=\sum_{x=k}^{m}\frac{\left(\begin{array}{c} M\\ x \end{array}\right)\left(\begin{array}{c} N-M\\ n-x \end{array}\right)}{\left(\begin{array}{c} N\\ n \end{array}\right)},$$

where *N *is the total number of promoters, *M *is the number of promoters with motif A in the region for motif A, *n *is the number of promoters with motif B in the region for motif B, *k *is the number of promoters for which both motif A and motif B were found in the respective regions, and *m *is the minimum of *M *and *n*. The value of each region-pair (pixel) in Figure 1B shows -log_10_*P*_*hg*_.

It is not easy to precisely evaluate the co-occurrence of overlapping motifs because of the correlation polynomial (see Chapter 8 in [46]); in other words, the occurrences of two motifs are not independent in the case that they overlap with each other. We counted such a case as a single occurrence rather than a co-occurrence. By doing so, *x *in the above formulae is underestimated, and therefore, *P*_*hg *_is overestimated, and thus the detected motif pairs are sufficiently significant.

### Clustering of motif matrices

Each motif used in this study corresponds to a weight matrix in TRANSFAC, which is known to be redundant [47,48]. Thus, we clustered motifs into a non-redundant set. As a distance measure, we used the averaged Kullback-Leibler distances (AKLD) per site over the aligned length between the corresponding positions of two matrices (we chose the alignment between the two matrices that gave the smallest distance). With the threshold of AKLD set to 0.5, the 358 matrices were clustered using the single linkage agglomerative algorithm; as a result, 228 clusters were obtained.

### Classification of different peak types of single motifs

Each single motif was classified based on the shape of its positional distribution. First, motifs with less than 30 occurrences were assigned to the 'no data' (ND) category. Second, the promoter region [-2000, +1000] was divided into bins of length 100 bp. Third, for each motif, its occurrence at each bin and each strand (plus or minus) was counted for all promoters. Fourth, the average values and standard deviation (SD) were calculated for all bins and strands except the region [-300, +300]. The positional distributions of all motifs were checked by eye to ensure that all simple peaks existed within the range. This region was determined such that no obvious peak covering the core promoter in frequency was observed outside of the regions in the positional distributions of all motifs. Finally, if the frequency of the motif in a bin within the region [-300, +300] was equal to or larger than the average plus SD multiplied by 2 (or 1), the motif on the strand was classified as a large peak (LP) type (or the small peak (SP) type); otherwise, it was classified as a no peak (NP) type. Because clusters of motifs (see above) may have members that are assigned to different peak types, we assigned a peak type to each cluster, as follows. For peak types LP, SP, NP, and ND, we assigned numbers 4, 3, 2, and 1, respectively. For each cluster, we then calculated the average of the values of its member motifs. The cluster was then assigned to the peak type closest to the obtained mean value.

### Classification of motif pairs

Motif pairs were classified into three classes using the following variables and the procedure shown in Figure 2. To characterize the patterns observed in the heat maps (e.g., Figure 1), 'high-scoring' regions (region-pairs) were defined. In this definition, the 'score' means the 'heat', and the 'high score' means that the score is no less than a threshold value, *Th*_*raw *_or *Th*_*mlp*_, in characterizing the raw count map (the negative log-p map). *Th*_*raw *_is defined as *Th*_*raw *_= *S*_min _+ (*S*_max _- *S*_min_) × 0.6, where *S*_max _(*S*_min_) is the maximum (minimum) score in the region. In characterizing the raw count maps (Figure 1A), two additional values were defined: the TSS-ratio (rtss) and the diagonal-ratio (rdiag). The TSS-ratio is the ratio of the number of high-scoring region-pairs in [-500, +500] to that in the entire high-scoring region, while the diagonal-ratio is the ratio of the number of high-scoring region-pairs in the diagonal region (i.e., the distance between the two bins is less than 500 bp) to that in the entire high-scoring region. Similarly, in characterizing the negative log *p*-value map (Figure 1B) we defined the TSS-ratio (htss) and diagonal-ratio (hdiag), although in this case we defined the threshold as *Th*_*mlp *_= *S*_min _+ (*S*_max _= *S*_min_) × 0.9.

### Expression analysis of genes

As a measure of the anti-tissue-specific expression of a gene, we used the number of EST libraries corresponding to the gene (UniGene cluster) in the NCBI UniGene database [16]. To reduce the background count of motif pairs in non-significant region-pairs, we selected the region-pairs where the scores are more than 2/3 of the most significant region-pair in the second heat map (Figure 1B) for each motif-pair. For each gene, we obtained the number of motif pairs subject to each class. The numbers indicate the extent how the gene is correlated with the Class l. The numbers were used both for identification of gene groups for each class, and for calculation of correlation coefficients.

### Domain analysis

For all 228 motifs described in the subsection "Clustering of motif matrices", we retrieved the corresponding TFs and their functional domains annotated in the TRANSFAC database. We obtained *F*_*d *_= 188 TFs with at least one identified domain. For each domain, we also calculated the number *F*_*c *_of TFs containing it. For each of the three hub-TFs groups (class1, class 2 and total) reported in Table 1, we constructed a hub-network comprising all the hub-TFs of a group as well as all the TFs pairing with them. We used odds-ratios to determine whether or not a given domain was enriched within a hub-network. For this, we calculated the number *F*_*a *_of occurrences of TFs with a given domain in the hub-network, and the number *F*_*b *_of occurrences of TFs with any domain in the same hub-network. Domains represented by less than 3 TFs, or for which the ratio *F*_*a*_/*F*_*b *_was lower than 0.1 were removed, and the odds-ratios *F*_*a*_/*F*_*b*_/(*F*_*c*_/*F*_*d*_) were calculated. P-values were calculated for domains with an odds-ratio bigger than 1.5 using a binomial background model in which the number of successes, trials, and the success rate were *F*_*a*_, *F*_*b *_and *F*_*c*_/*F*_*d*_, respectively. The results are shown in Table 2.

## Authors' contributions

KM designed the analysis, developed the algorithms, and wrote the manuscript. TI and TG participated in the design and execution of the study. KN participated in the design of the study and wrote the manuscript. All authors read and approved the final manuscript.

## Supplementary Material

Additional file 1 Complete list of significant motif pairs. Click here for file

Additional file 2 Plot of the degree (the number of motif counterparts) versus rank of motifs involved in the motif pairs in Classes 1 and 2. In principle, those motifs with higher degrees (left-hand side in each plot) can be regarded as hub-motifs. For convenience, the top 20 motifs were chosen as hubs. The distinct distributions of the two classes indicate the different usage of their motif members. Click here for file

Additional file 3 Log-log plot of the degree (the number of motif counterparts) of motifs and their frequency for each degree. The network was constructed from the top 600 significant motif pairs, consisting of 88 motifs. The linear relationship suggests that the network is scale-free. A value of Log(k) of n represents a value of k of 10^n^. Click here for file
