# Supplementary figures and images for: Two different classes of co-occurring motif pairs found by a novel visualization method in human promoter regions
Source: BMC Genomics. 2008 Mar 1;9:112. doi: 10.1186/1471-2164-9-112 (PMC2292176; doi:10.1186/1471-2164-9-112)

## Slide 1
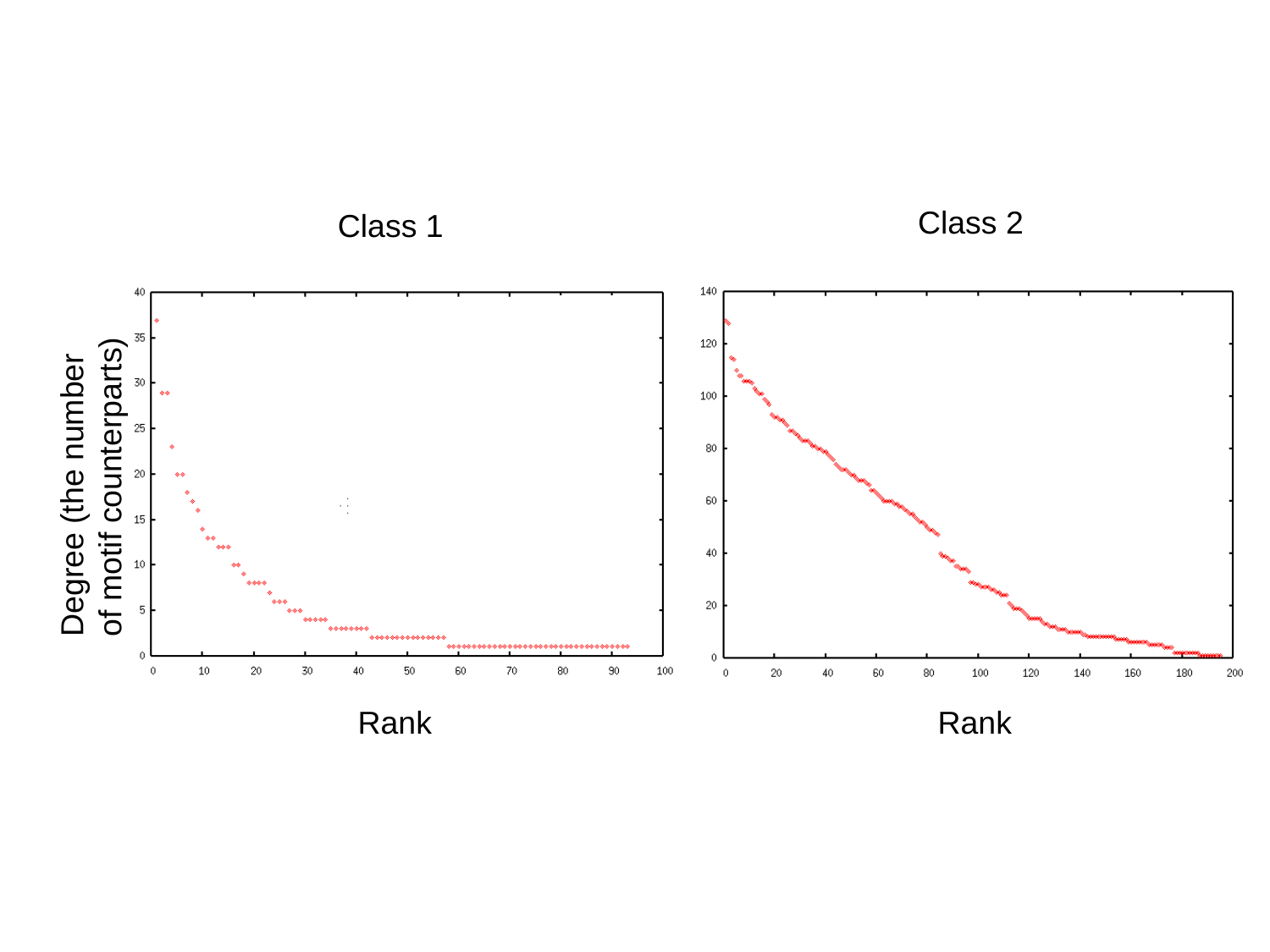

Class 2
Class 1
Degree (the number
of motif counterparts)
Rank
Rank

Supplement: Additional file 2 — Plot of the degree (the number of motif counterparts) versus rank of motifs involved in the motif pairs in Classes 1 and 2. In principle, those motifs with higher degrees (left-hand side in each plot) can be regarded as hub-motifs. For convenience, the top 20 motifs were chosen as hubs. The distinct distributions of the two classes indicate the different usage of their motif members. [file 1471-2164-9-112-S2.ppt]

## Slide 1
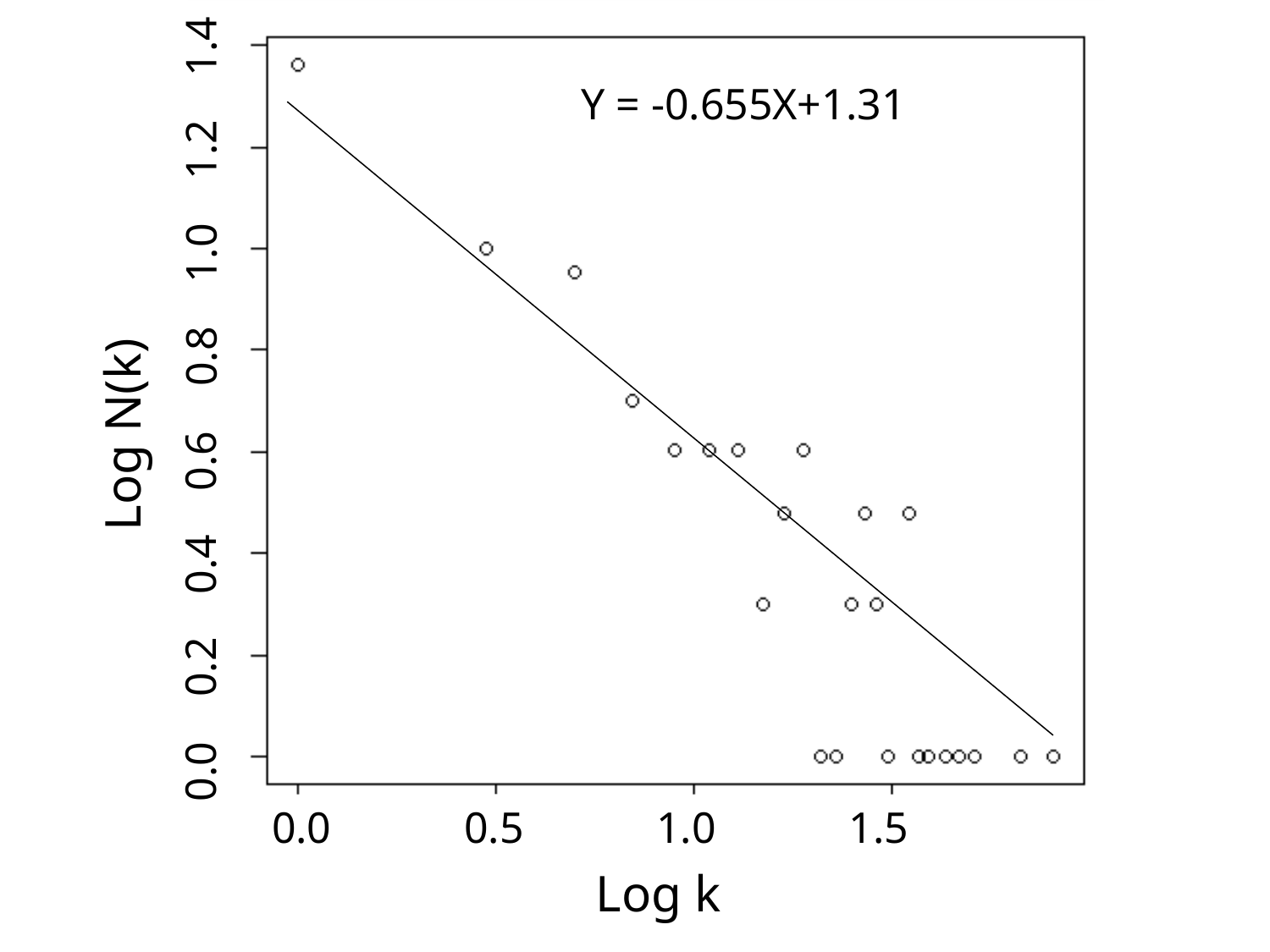

1.4
1.2
1.0
0.8
0.6
0.4
0.2
0.0
Y = -0.655X+1.31
Log N(k)
0.5
0.0
1.0
1.5
Log k

Supplement: Additional file 3 — Log-log plot of the degree (the number of motif counterparts) of motifs and their frequency for each degree. The network was constructed from the top 600 significant motif pairs, consisting of 88 motifs. The linear relationship suggests that the network is scale-free. A value of Log(k) of n represents a value of k of 10n. [file 1471-2164-9-112-S3.ppt]
